# Factors Associated with Prehospital Delay among Men and Women Newly Experiencing Acute Coronary Syndrome: A Qualitative Inquiry

**DOI:** 10.1155/2020/3916361

**Published:** 2020-05-11

**Authors:** Lourance A. Al Hadid, Marwa Al Barmawi, Nathira Abdelqader Ahmad Al Hmaimat, Noordeen Shoqirat

**Affiliations:** ^1^Department of Nursing, Aisha Bint Al Hussein College of Nursing and Health Sciences, Al Hussein Bin Talal University, Ma'an (71111), P.O. Box: 20, Jordan; ^2^Department of Nursing, Faculty of Nursing, Alzaytoonah University of Jordan (ZUJ), Amman, Jordan; ^3^Fatima College of Health Sciences, Abu Dhabi, UAE; ^4^Nursing Faculty, Mutah University, Karak, Jordan

## Abstract

**Background:**

Delaying seeking health care for patients with acute coronary syndrome (ACS) causes high mortality and morbidity with variations among men and women regarding reasons for this delay.

**Objectives:**

This study explored factors associated with prehospital delay among men and women experiencing acute coronary syndrome for the first time in Jordan.

**Methods:**

35 men and 33 women with ACS admitted and treated at the coronary and postcoronary care units.

**Results:**

Themes emerging from the data are knowledge about ACS, the resources related to health care, and concerns around family wellbeing. Owing to the traditional roles of men and women within the family, women felt responsible for maintaining the family, assisting in the financial conditions, and supporting family coherence by delaying hospitalization. Men were worried about the structural safety and maintenance of the family. *Conclusion and Implications*. Prehospital delay is common among first-timer ACS patients from both sexes, and thus, increasing awareness about ACS among the public from all age groups is necessary. Availability of specialized health care centers and equity in health care services are vital to improve public confidence in these health care settings and health outcomes.

## 1. Introduction

Acute coronary syndrome (ACS) is a common cardiovascular disease that includes a number of conditions affecting adults from both sexes. These conditions are unstable angina, ST-segment elevation myocardial infarction (STEMI), and non-STEMI [1]. The ACS contributes to high mortality and morbidity rates leading to major defects in the life style of individuals and families [2]. It is one of the main causes of death among adults in many countries around the world, including Jordan [3, 4]. Although the prevalence of ACS among men is higher than women, the incidence of this syndrome increases two or more folds in women after menopause, which necessarily indicates the impact on both sexes in different age groups [5]. Many studies have investigated sex disparity for patients with ACS in treatment outcome and delayed admissions due to alert–activation related to symptoms and ST elevation [6]. However, limited reports focused on reasons causing prehospital delay of individuals experiencing ACS from both sexes in Jordan and the region, which has been reported to worsen the prognosis leading to higher morbidity [5].

Women continuously show increased tendency to delay seeking health care as compared to men for various reasons [7, 8]. For instance, women can experience prehospital delay due to the assumption that they have a low probability to develop ACS and therefore may view this condition as men-specific [9]. Women may also experience delay due to variations in the symptoms, which can be confused with gastrointestinal tract or common cold symptoms [10].

To date, the growing literature reported that prehospital delay had caused serious complications in both sexes, such as heart failure and even death [11]. However, similar studies were not located in the Middle East in general and Jordan in particular, which has been reported to have a high prevalence of ACS among different age groups [3, 4]. The existing body of evidence reflects mainly Western Countries, such as Australia, the UK, and North America [2, 12, 13], and its applicability to Jordan can be questioned. Therefore, this study explored factors associated with prehospital delay among men and women experiencing acute coronary syndrome for the first time in Jordan. It is hoped that the emerging data might be used as a springboard that guides devising future educational programs and policies for the public, including patients with coronary artery diseases.

## 2. Methods

### 2.1. Design

A qualitative design using face-to-face, semistructured interviews was adopted with patients admitted and treated for the first time for ACS at the coronary and postcoronary care units. All study methods and results conform to the COREQ-checklist for qualitative studies [14]. Qualitative interviews, deemed appropriate for this study, allowed the researchers to follow interesting leads within the narratives. Using semistructured interviews, participants explained their experiences in their own words; therefore, unexpected and interesting themes appeared during the interview process [15]. Semistructured interviews are valued as a method that allows flexibility for the interviewer to clarify responses and ask probing questions thereby providing opportunity for better exploration of experienced events [16].

### 2.2. Settings and Study Sample

Candidates were patients diagnosed with ACS and receiving treatment (during data collection) at one of the four hospitals included in the study. Inclusion criteria were as follows: patients were 18 years old and above, experienced ACS for the first time, could speak Arabic or English, were conscious and oriented, and had stable vital signs (heart rate, blood pressure, temperature, respiratory rate, and pain) for the past 12 hours and more. Exclusion criteria included patients, who were previously diagnosed with ACS, unconscious, sedated, or experiencing unstable vital signs. Patients with any mental or psychological instability were not included in this study. Additionally, pregnant women were excluded from the study.

### 2.3. Data Collection Procedure

The researchers and the charge nurses worked collaboratively to identify and recruit candidates for the study. Potential candidates diagnosed with ACS were assessed based on the inclusion and exclusion criteria for participation. The study was conducted on patients admitted to four hospitals in Amman (the capital city) and the governorate of Ma'an at the South of Jordan, two hospitals from each area. The researchers approached the candidates and asked for their interest in being part of the study. As candidates expressed interest, the study objectives and procedure were explained fully by one of the researchers. Semistructured interviews enabling both standardization and flexibility were used to collect the data between February 2019 and June 2019.

There were 37 men and 41 women, who expressed their willingness to participate in the study. However, 35 men and 33 women completed the study interviews. Some patients (*n* = 10) did not complete or refused to participate in the interviews for different reasons, such as planning to transfer to another unit, or feeling unwilling to participate, or changing their decision and refusing without expressing reasons. None of the patients, who did not participate, expressed their decision because of reasons related to the study.

The delay time in this study was defined as the time between the beginning of chest pain feeling and asking for help, whether professional via phone or asking a family member to call for a professional medical help. All data concerning the delay were taken directly from the patients.

Interview questions were adopted from the published international literature [17] covering themes frequently reported to influence the decision to seeking health care, and were associated with prehospital delay. Interviews included questions and probes as follows: “I would like to learn about what happens when you started to feel the symptoms,” “what and when did you seek help as symptoms were present?”, “what thoughts came to you when you were experiencing the symptoms?”, “how do you believe your condition would be affected when going back home?” More questions were used in the interview as it progressed adding further to the process of exploring the phenomenon and depending on the personal engagement of the interviewee [18].

All interviews were conducted by the first and the second authors (LH and MB) in the coronary and postcare units within two periods of time during the day. The first period was between 9.30 am and 12.30, and the second period 15.30 and 18.00; no night times were included in data collection. During those times, there were very low level of noise and almost no visits, thus providing acceptable levels of collaboration and willingness of patients to engage in the interviews. As the interview commenced, participant's privacy was preserved by closing the curtains or doors. No third person attended or listened to the interview. The researchers consciously decentered their perceptions, assumptions, and ideas taken for granted while conducting the interviews to ensure that the participants' experience was reflected without any contamination [19]. Interviews were digitally recorded and then were fully transcribed verbatim by the researcher, who conducted the interview, and then transcripts were checked by another researcher for clarity.

The interviews lasted between 42 and 56 minutes to complete excluding the introduction and voice testing the audio-recorder. Transcripts were returned to participants to approve within 24 hours of the interview. All interviews were conducted in Arabic and then transcripts were translated into English by a professional English translator after being approved by the participants in its original Arabic version.

During the conduction of the study, the interviewers were attentive by providing a constant reflection and clarification to ensure that participant's understanding of the interview questions was precise and to give a sense of livelihood to the interview, thus minimizing feelings of boredom [20].

### 2.4. Ethical Considerations

The researchers obtained the IRB approval from the Ministry of Health to collect data from the intended hospitals (no. 26/14/2018–2019). All patients signed a consent indicating that all information concerning the study purpose and procedure were explained fully and that they were willing to participate under no pressure or coerciveness from anybody. The researchers further emphasized that all information provided by the participants would be treated confidentially and should not at any time reveal the identity of the provider. The consent also has a section that declare using a digital audio-recording to the interview (without identifying the participant), and that they could withdraw from the study at any time. The researchers ensured all participants that they could stop the interview if they felt uncomfortable or that there were inappropriate issues about the procedure.

### 2.5. Data Analysis

The interviews were conducted using the Arabic language as all patients were native Arabic speakers. All interview recordings (*n* = 68) were translated from Arabic into written English texts following a strict method to ensure the reflection of accurate meanings. Therefore, the following measures were adopted in this process: audio-recordings were listened to attentively and matched with the corresponding transcript, which were then translated by the researcher, who conducted the interview (namely, LH and MB); the researcher asked another researcher to check the transcriptions against the original recordings of the interview audio data [21]; and then each translated version was investigated by all authors and any difference was discussed thoroughly to ensure the best word and meaning choice by reexamining the recorded interview. After a consensus had been reached among all authors, all transcripts were submitted to analysis.

The researchers in this study used inductive content analysis to analyze the interview transcripts [22]. The researchers used open coding to identify themes that were common among men and women, and then subthemes were arranged into categories as they were bundled into categories. Each transcript was separately inspected and then joined for inspection together. Each transcript was examined line-by-line to identify all ideas and patterns, which were bundled to formulate categories. These categories formed the themes and subthemes, which were checked by all authors to approve.

### 2.6. Rigor

Credibility of the data interpretation was achieved by logically establishing the research method and audio-recording the semistructured interviews [15]. Within the cultural norms, patients used their own words to explain their opinions during the semistructured interviews; these were then used as quotations when writing up the analysis findings. In addition, interpretive validity was ensured by representing the meaning and the views of the participants, who represented the insider perspective and the fact that the interviewers did not impose their own perspectives [23]. Confirmability has been established by being clear and objective when conducting the interviews, documenting and managing the audio-recorded data, reporting the research process and the findings, and drawing conclusions based on these findings [24].

Although the contribution of the study can be determined by the authors, deciding the transferability or ‘fittingness' of findings to other settings is the responsibility of those who will be using these findings, not the researchers [25]. However, the researchers in this study took all necessary steps to ensure the transferability, including the recruitment of participants purposefully to represent a variety of different circumstances, providing rich contextual data and promoting better representation of individuals comprising the situation under investigation. The researchers also visited the clinical areas and talked to patients, who represented different age groups, educational levels, sexes, and areas of resident. This process resulted in a mix of participants, who represented a range of personal characteristics.

## 3. Results

### 3.1. Characteristics of the Participants

The age of participants ranged between 22 and 74 years for both sexes with men relatively younger than women (Table 1). The average age for men was 42.3 years and was 52.6 years for women. Men had relatively higher academic degrees as compared to women. All men and the majority of women were married. Almost all men were either self-employed or employed, and more than half (*n* = 25) the women were nonworking housewives or retirees. The comorbidities among women were more prevalent than men, including diabetes, hypertension, and hyperlipidemia.

There was a significant difference in prehospital delay among both sexes; men delay time is significantly longer than women.

Men and women reported various sites of chest pain, including localized chest pain, shoulder pain, numbness of the upper limbs, and less frequently stabbing, and heartburn (Table 2). The majority of men and women in this study reported experiencing chest and shoulder pain as a result of ACS (28 and 26, respectively). Younger men reported having a stabbing chest pain more often than women. Generally, participants reported having different symptoms included in Table 2.

While many participants in this study reported experiencing no similar symptoms, others reported having less intense symptoms during the last twelve months (Table 3). Among all participants, only 7 participants (3 men and 4 women) said that they sought medical advice when symptoms recurred. However, none of the participants was previously diagnosed with ACS. The onset of symptoms differed among participants from both sexes with many reporting the midday to afternoon hours to have the highest prevalence, especially men. Interestingly, a relatively high proportion of participants from both sexes reported experiencing prehospital delay. They also said that they were not aware of ACS symptoms.

The majority of participants, from both sexes, reported that they were not aware of the ACS symptoms or they did not believe those symptoms were related to a cardiac condition. While health insurance and health care cost were mentioned as the main concerns, family appeared to be in the mind and heart of the majority of participants. Although many similarities between men and women can be noticed in the findings, some differences are still noticed, especially those concerned with the role within the family. The thematic analysis of participants' narratives identified factors associated with the delay in seeking health care (Table 4).

### 3.2. Theme One: *Knowledge about ACS: What Is True and What Is Not?*

Participants (*n* = 58) reported not knowing that the symptoms they experienced were related to a cardiac problem coupled with some misconceptions, including mixing ACS symptoms with those related to indigestion and common cold. As evident by data this is because ACS symptoms might range from simple flu-like, nausea, and abdominal pain to severe stabbing chest pain. The majority of participants (25 men and 23 women) reported not knowing the symptoms of ACS, or even receiving any education about them.

*Really, I barely heard about ACS. In fact, I did not know what a person might experience during the attack. I was not sure this pain is caused by a heart problem. In fact, I still cannot believe it is my heart. I think it is my stomach. I have always had issues with my stomach.* H3M5.

Although the prevalence of ACS in Jordan is relatively significant, many participants (*n* = 59) stated that they did not receive any form of education about emergency cardiac conditions, including ACS. The above quote has been taken from the interview of an educated woman with a degree in Arts. It might be assumed that this educated woman might have heard about heart attacks (i.e., ACS) during her academic study or work. However, this did not appear among participants in this study. Another male participant attempted to link his signs and symptoms to the unhealthy lifestyle practice as illuminated below:

*I smoke packed cigarettes, nearly 40 per day. So, I thought it was my chest. Therefore, I took a hot drink and went on to my work. But the pain kept increasing. It never came to my mind it was my heart. I'm young and not supposed to have a heart problem.* H1M5.

The above evidence would imply that the lack of knowledge about the symptoms ACS affected participants' actions. For instance, despite the fact that the participant was aware of a possible cardiac problem, he only took a hot drink and continued working instead of seeking medical care. Furthermore, it has been frequently indicated by participants in this study that heart disease can only be found among the elderly:

*I know that heart problems come with aging. Right! My father-in-law, a 78-year-old man, had problems with his heart. My aunt also was above 60 years of age when she had problems with her heart. I did not imagine that I will have similar problems with my heart.* H2M3.

Another false assumption related to referring to ACS as stomach or GIT related symptoms appears in the following quote:

*I had a heavy meal the night before the heart attack. I spent that night trying to sleep, but my stomach was aching. I did not have this feeling before. So, I thought it was only my stomach. I took a tablet for my heart burn and ache. I did not think for a minute it was my heart, and not my stomach.* H4F2.

The above data would suggest that the lack of knowledge about ACS coupled with personal misconceptions played a vital role in delaying the counseling and thus seeking help. This is an important issue to keep in mind hence by doing so a serious complication or life-threatening status might be the outcome.

### 3.3. Theme Two: *Poor Resources and Lack of Trust*

Further analysis of qualitative data uncovered another important contributing factor to the delay of seeking medical help. The availability of health insurance and health care professionals, who could provide emergency health care when needed, is a frequently found obstacle in the transcripts. Women were more concerned about the availability of nearby qualified health:

*I could not think of the nearby hospital. I cannot trust them. We don't have a center that provides good care when we have life-threatening situations, like mine.* H4F7.

The above evidence is confirmed further by a number of participants (*n* = 32), mainly from rural areas, who reported having no confidence in the nearby hospitals or doctors due to a number of previous negative experiences with close relatives.

*I do not trust the nearby hospital. The staff there are not very skillful. We had many incidences when people entered and died. Nobody gave us answers why my mother died five years ago.* H4F5.

Rural-living patients had serious concerns about the availability of well-trained staff to provide safe and appropriate care to them and their patients. This concern did not appear in the interviews of those living in urban areas. As the absence of a health insurance poses a serious problem to some, participants reported their concern about the cost of health care services. This is particularly reported by men.

*It is true that health services are not so expensive, but we still have to pay a lot of money. I wish to have a health insurance that can cover my health care services so that I can feel much better without being concerned about the cost of each procedure the doctors and nurses do for me.* H1M6.

In addition to the unpleasant experience, some patients reported having concerns they might suffer serious complications, including major disability and death. Participants were afraid they might die because of negligence or due to incompetent or inappropriate doctor decisions:

*I still remember the death of my brother. He was in a good health. He only had abdominal pain when we brought him. Two hours later, they came out of his room telling us that he died. It turned out later that they did not know what was wrong with him. I don't want to have a similar experience. I'm counting hours to go out.* H4M2.

Other reasons, like residential area (urban, suburbs, and rural areas), caused differences in the decision to seek health care among ACS victims. Some participants related variations in the quality of health care services to the availability and accessibility of the proper resources, like personnel and equipment.

*My mother needed a cardiac catheterization. We had to move her more than 200 kilometers to the nearest hospital to do cardiac cath. It was not a very nice experience at all.* H3M3.

Those comments were mainly from participants living in suburban or rural areas. In addition, although participants had health insurance that covered the cost of health care, another worrying issue was expressed by patients coming from rural areas. They were concerned about the cost and the financial burden caused by traveling of a family member to stay in another city with their patient during hospitalization.

*As you know, I live in* [name of the village]. *Therefore, my husband and children would leave all their business and stay with me to make sure I don't need anything. This would take them to rent a room and pay for their food and the drinks during my stay, and this is really very expensive. Although it is very difficult, but I hope we would manage.* H3F9.

Patients emphasized on the presence of variations in the quality of health care and a concern about the costs of health services as well as the expenses of their stay if they had to move to another city for treatment. Resources, personnel, and equipment together coupled with lack of trust were among the major issues addressed by many patients in this study causing significant prehospital delay among men and women, and in particular patients coming from rural areas.

### 3.4. Theme Three: *My Dependent Family Members Are a Significant Concern to Me*

Participants in this study expressed their concern regarding family wellbeing after experiencing ACS. Participants repeatedly expressed that they were thinking of their family dependents and how they could manage if anything bad happened to them, like death. However, a closer analysis showed that there has been a difference among patients from both sexes. Men reported concerns regarding the family maintenance and financial competence.

*You know, I was thinking during the attack. If I had a serious condition, like death, what would happen to my children? No one would support them. I tried then to take any medication to help me sleep. I did my best so as not to go to the hospital. With me working and earning a living for them, it is still very difficult. These are the kind of questions I had during the pain attack.* H1M8.

Women, on the other hand, had different concerns and said that the main thought was about who would take care of their children if anything wrong happened to me. Only a few women were worried that their husbands would remarry, and they were concerned about their school-age children wellbeing. The low number of women reporting this issue (*n* = 8) could be related to not knowing that they had ACS, a life-threatening condition.

*I was thinking that I might die and my husband would marry another one. My kids are still very young; they are between 4 and 12 years of age. It was a terrible experience. What would happen to them if I die?* H4F4.

Participants tried to abstain from thinking that the pain attack was related to a serious cardiac condition, which, in some cases, could lead to death. Therefore, some were discouraged from seeking help.

*During the attack, I had a strange feeling. I thought that I might die and leave my kids and husband. They are my life, you know. I did not want to come to the hospital. I took medication to minimize the pain, and I tried to stay calm by not telling anyone that I had this severe pain. But finally, I could not take it anymore. Oh, my husband is a very warm person. He is now taking care of my kids. Thank goodness I did not die and leave them to suffer afterward*. H2F5.

As appeared from the narratives, men and women had many common themes but with some differences in the details. Perhaps one of the main issues that caused these differences was the different social roles and expectations carried out both in the family and in the society.

Although different roles created different views and concerns to the same problem, also the residential area and confidence in the available resources (such as trained personnel and diagnostic equipment and procedures) contributed significantly to the decision to delay seeking health care.

## 4. Discussion

There are many studies reporting on differences between both sexes regarding the physiology, symptoms, and mortality [6, 8, 26]. This study adopted face-to-face interview to report differences between men and women in terms of reasons they believed were associated with prehospital delay when experiencing ACS symptoms for the first time.

Findings in this study indicate that ACS patients' prehospital delay can be due to lack of knowledge about the ACS symptoms, inability to interpret ACS symptoms, or confusion between ACS and other symptoms related to the upper respiratory or gastrointestinal tracts. A number of studies indicate similar findings like lack of knowledge about ACS, misinterpretation of symptoms, lack of health insurance, and accessibility to health care [2, 27, 28]. However, the emerging data in this study contribute to the existing empirical literature by highlighting differences in the roles among men and women within the family and the society. The Jordanian society has its unique elements of modernism, not so conservative, but not so liberal. This means that it is mainly a civilized society with a very high percentage of education, ruled by a legal system and respects women and men responsible freedom. Women in this society can go out, drive a car, work, and make a social network, but within an eastern customary system that is neither unrestricted, nor very conservative. Therefore, women in Jordan are equal citizens with all rights and obligations like men.

Men and women enjoy a degree of responsible freedom where both participate in the decisions concerning the family affairs and their future. Although women are men's partners in maintaining and supporting family members, women work more as the family internal affairs' officer, while men care more about the outside affairs. Men are usually responsible for providing the financial stability of the family, and equally important women run the family issues from taking care of the house to caring for the children ensuring that all members are well-taken care of, including the husband [29]. In other words, roles of men and women might differ from those in Europe, North America, and Australia. Women usually assume a more liberal role as partners in all aspects of life in these countries. However, women in Jordan are commonly responsible for the household or domestic roles and men are the main bread-winners responsible for the financial support of the family and ensuring its stability [30]. Although this is commonly found in the Jordanian society, other examples also exist, such as those families where men and women are equal partners [31]. Therefore, responses from men and women might reflect the assumed roles within the family.

Findings in this study confirm that the majority of women were concerned about the internal family issues compared to men, who expressed their worry regarding issues about family support and the financial adequacy. In addition, the prehospital delay could be affected by nonhealth related issues, like the educational preparation, the surrounding culture (like women do not develop acute heart diseases, such as ACS), and the social environment [7, 31, 32]. Studies [13] show that acknowledging cultural and social factors is essential to improve understanding of why and how the decision to seek health care is made when experiencing the symptoms.

Others [33] emphasize that differences are present between both sexes with regard to knowledge about heart disease. In this study, knowledge deficit was on top of the issues, but with almost no difference between men and women. Some argue that limitations in symptom recognition and interpretation and lack of autonomy might cause women prehospital delay where they feel dependent on men, like their husbands and grownup sons, to meet requirements outside their homes [7, 34]. Interestingly, the presence of comorbid condition did not show any impact on decreasing the time of delay improving patient response to symptoms. Research presents evidence suggesting failure to recognize ACS symptoms due to the nonspecific symptoms and sex presumptions that indicate ACS as a men-only condition [8, 35]. In addition, experiencing atypical symptoms, such as shoulder pain, nausea, and abdominal discomfort, both confuses the individual and results in higher rates of delay among both sexes.

The presence of atypical ACS symptoms, such as muscle aches, shoulder discomfort, and abdominal pain, may also result in a greater delay in seeking health care, perhaps as women may attribute this discomfort to digestion, to aging, or due to house work [36]. Therefore, ACS patients, especially women, misinterpret the meaning of their symptoms, the presence of ACS, and delay asking for health care [18]. Some studies [36, 37] found that young patients after ACS usually do not consider the presence of any cardiac condition based on the assumption that says “cardiac conditions are age-dependent conditions; the young adults are safe.” These studies further confirm findings from our study about the Jordanian patients.

Moreover, our data would postulate that the economic status was among the issues reported by participants in this study to influence the timeliness in seeking care. While men are usually the bread-winner in Jordan, women have recently become partners by providing financial support to the family. Some [38] argue that women might feel responsible for maintaining the family, assisting in the financial conditions and supporting the family coherence by delaying hospitalization and by using alternative therapies. Therefore, they delay asking for health care trying to avoid extra costs caused by hospitalization and moving next to the hospital, in particular for those living in rural areas away from the referral hospital [39]. Similar to these findings, a study from Iran [40] indicated that the long distance between residence and hospital was among the common causes of prehospital delay among ACS patients living away from hospitals.

In comparison with the above discussion and debate, men in this study were mostly worried about the impact of their absence or disability on the family economic and financial status.

Women, on the other hand, reported delaying asking for health care due to their concern about the wellbeing of the family wondering who would meet the needs of each member within the family when they are away. Although patients were concerned about the safety and integrity of the family, women were unselfishly worried about the integrity and wellbeing of the family. Owing to the traditional roles of men and women within the family, researchers [41] contend that understanding the social roles of each individual is imperative to explain the differences between both sexes. In Jordan, the man has the authority and is the bread-earner, while the woman runs the house. Therefore, findings in this study reflected similar issues usually seen in the society [42]. Acknowledging these differences should be present when developing programs addressing findings in this study.

## 5. Limitations

This study did not collect data from participants, who experienced previously similar symptoms.

In addition, this study included participants from two geographical areas, which could limit generalizability. This study did not explore in-depth factors that influenced ACS decisions, including social and cultural factors. Although it appeared very important in the results of this study, the impact of sociocultural factors was not fully addressed in this study given its qualitative nature. However, the emerging themes from the study might be used as a benchmark supporting future studies that would be developed investigating this unexplored area.

## 6. Conclusions

Participants from both sexes had a notably very limited knowledge about ACS. Men were concerned about the wellbeing and the financial support of the family, and women were more concerned about the dynamics within the family and more often reported their fear about who will take care of children. Therefore, patients from both sexes need to receive education that addresses role changes after ACS. Furthermore, patients from both sexes, especially those living in remote and rural areas, reported concerns regarding the availability of resources, such as cardiologist and trained nurses. Equity in services and resources is important to achieve the national objective of health services to all, which is the slogan usually portrayed by the government. Future research might address these issues to identify approaches that highlight optimal health care, new roles after ACS, and strategies to adapt to after ACS social life. Understanding and tackling factors associated with prehospital delay are important to decrease both mortality and morbidity from ACS in Jordan. This is particularly of significance to the national medical profile in Jordan where the age of those experiencing ACS is relatively low compared to published international figures.

## Figures and Tables

**Table 1:** Characteristics of the study sample (*n* = 68).

Factor		Men (*n* = 35)	Women (*n* = 33)
Age	20–30	4	2
	31–40	12	1
	41–50	13	5
	51–60	4	11
	61–70	2	3
	>70	—	11

Level of education	High school	3	10
	Diploma	8	6
	Bachelor	19	16
	Masters-Ph.D.	5	1

Work	Housewife	—	23
	Retired	2	2
	Currently employed	14	5
	Self-employed	19	3

Comorbidities	Hypertension	4	8
	Diabetes mellitus	5	14
	Hyperlipidemia	8	15
	Heart failure	1	3
	Hypothyroidism	1	1

Prehospital delay time (hours)	Range	1.5–24	1–2.5

Some responses were approximate.

**Table 2:** Prevalence of symptoms related to acute coronary syndrome.

Site	Men	Women
Chest pain	28	26
Shoulder pain	14	19
Stabbing pain	14	11
Numbness in shoulders and arms	12	16
Flu-like symptoms	12	9
Heartburn	11	10
Dizziness	3	12

**Table 3:** Timing and history of the ACS symptoms.

		Men (*n* = 35)	Women (*n* = 33)
History of having similar symptoms	Yes	16	8
	No	19	25

Timing	Early morning	5	4
	During the day (with minimal effort)	2	11
	Afternoon	21	4
	Evening	—	5
	During the night	7	9

Delay seeking a specialized (professional) care	Yes	28	20
	No	7	13

Were you aware of the ACS symptoms	Yes	5	7
	No	30	26

**Table 4:** Themes reflecting reasons for prehospital delay among ACS patients.

Theme	Subthemes	Men (*n* = 35)	Women (*n* = 33)
Knowledge about ACS: what is true and what is not?	Nobody knew about ACS symptoms	25	23
	I couldn't believe I had a cardiac problem as I'm still young	22	19
	I believed than it was my stomach, but not my heart	15	18

Poor resources and lack of trust.	I don't have any health insurance coverage	21	9
	Health care cost is very high and I can't afford it	19	12
	We don't have a health professional around to assist when we need help	13	19

My dependent family members are a significant concern to me.	Who will support my family if I become disabled or even die?	27	—
	Who would take care of my family?	11	8

## Data Availability

Permission to make these data available for public use has not been granted by the participating patients. Therefore, we cannot make them publicly available.
